# Upregulation of Canthaxanthin Biosynthesis by *Paracoccus bogoriensis* PH1 from Hot-Spring Origin via Sustainable Fermentation Strategy in Laboratory-Scale Bioreactor

**DOI:** 10.3390/biology14101334

**Published:** 2025-09-27

**Authors:** Anuttree Inyoo, Phitsanu Pinmanee, Paweena Thongkred, Kanok Wongratpanya, Amonrat Kanokrung, Rawiwan Watanadilok, Jeeraporn Pekkoh, Chayakorn Pumas, Pachara Sattayawat, Sakunnee Bovonsombut, Wasu Pathom-aree, Thidarat Nimchua, Thararat Chitov

**Affiliations:** 1 Department of Biology, Faculty of Science, Chiang Mai University, Chiang Mai 50200, Thailand; anuttree_inyoo@cmu.ac.th (A.I.); jeeraporn.p@cmu.ac.th (J.P.); chayakorn.pumas@cmu.ac.th (C.P.); pachara.sattayawat@cmu.ac.th (P.S.); sakunnee.b@cmu.ac.th (S.B.); wasu.p@cmu.ac.th (W.P.-a.); 2 Doctor of Philosophy Program in Applied Microbiology (International Program), Faculty of Science, Chiang Mai University, Chiang Mai 50200, Thailand; 3 Enzyme Technology Research Team, National Center of Genetic Engineering and Biotechnology (BIOTEC), Pathum Thani 12120, Thailand; phitsanu.pin@biotec.or.th (P.P.); paweena.tho@biotec.or.th (P.T.); kanok.won@ncr.nstda.or.th (K.W.); 4 Institute of Marine Science, Burapha University, Chonburi 20131, Thailand; amonrat_@go.buu.ac.th (A.K.); rawiwan@buu.ac.th (R.W.); 5 Environmental Science Research Center (ESRC), Chiang Mai University, Chiang Mai 50200, Thailand

**Keywords:** bacterial pigment, *Paracoccus*, extremophile, fermentation, functional ingredients, antioxidant

## Abstract

Canthaxanthin is a significant carotenoid that is synthesized by specific microorganisms. It has multiple functions and has been utilized in food and feed supply chains. This research focused on improving canthaxanthin production by *Paracoccus bogoriensis* PH1, an orange-pigmented bacterium isolated from hot springs. Canthaxanthin production was optimized in flask-scale cultures by varying the pH, temperature, nutritional sources, aeration rates, and agitation techniques. Flask culture cultivation indicated that canthaxanthin production by this strain was influenced by pH stress mechanisms, resulting in the establishment of a two-stage pH control (pH-shift) technique to enhance cell mass and pigment production. The optimum flask conditions were refined for application in a 1 L bioreactor. An optimized cultivation procedure was established utilizing a Polypeptone Sucrose Yeast Extract (PPSYE) medium, with a pH transition from 7 to 11, incubation at 40 °C, agitation at 250 rpm, and aeration at 2 vvm for 48 h. This process resulted in a 3.12-fold increase in total carotenoid content and a 1.61-fold increase in canthaxanthin production, achieving 0.84 ± 0.06 mg/L compared to pre-optimized flask cultures in TSYEB medium (pH 7 at 37 °C, 72 h). Purified canthaxanthin from *P. bogoriensis* PH1 exhibited antioxidant activity in the ABTS assay.

## Simple Summary

This study presents a systematic approach to optimize growth and enhance canthaxanthin pigment production by a hot spring dweller, *Paracoccus bogoriensis* PH1. Various parameters affecting pigment production by this bacterial strain were first optimized at the flask scale and subsequently at the 1 L bioreactor scale. A novel culture medium was developed, and an optimized process was designed using a pH-shift strategy with appropriate temperature and aeration rate. Purified canthaxanthin exhibited antioxidant activity, as determined using the ABTS assay. The optimized conditions identified in this study can potentially reduce the risk of contamination and energy demands for cooling, thereby offering an advantage for the sustainable industrial production of canthaxanthin from natural sources.

## 1. Introduction

Many carotenoid pigments are recognized for their biological activities, including antioxidant, antibacterial, antiviral, anticancer, and anti-inflammatory properties. Therefore, they have many applications in food and feed supplement supply chains and hold significant commercial value. Examples of major carotenoids with commercial significance include astaxanthin, canthaxanthin, lutein, and β-carotene. Astaxanthin is extensively used in the cosmetic industry and as a food and feed supplement [1,2] because of its high antioxidant activity. Canthaxanthin is used as a colorant in foods and beverages [3] and is indirectly used to enhance the color of aquatic animal species via animal feed [4]. It is also used in cosmetics and nutraceuticals [5,6]. Lutein is widely used in the food supplement sector, especially for preserving and promoting eye health [7]. Beta-carotene, a precursor of vitamin A [8], is primarily used to supplement vitamin A in the human body. Additionally, it is used to address vitamin A deficiency, which may result in visual impairment and dermatological issues. It is also used to manage erythropoietic protoporphyria (EPP), a rare genetic disorder characterized by photosensitivity in affected individuals.

Many carotenoid compounds, including canthaxanthin, can be chemically synthesized or obtained from natural sources. Nevertheless, there is a growing interest in the production of natural carotenoids from microorganisms [9]. The commercial value of the natural carotenoid segment was estimated at 1.35 billion US dollars in 2024 and is expected to reach 3.3 billion US dollars by 2035, reflecting a compound annual growth rate (CAGR) of 8.7% during the 2025–2035 forecast period [10]. Using microorganisms as commercial natural carotenoid sources has advantages over using plants and animals because of food security [11]. Considering the challenges of food security under climate change, the production of natural products, including carotenoids, using microbial technology offers a sustainable alternative to conventional agriculture for addressing food system challenges [12,13]. Bacteria are considered an alternative microbial source for pigment production because their cultivation conditions are easy to control, are not restricted by seasonal changes, and have a short cultivation period [14]. Natural bacterial sources of canthaxanthin include members of the genera *Gordonia*, *Micrococcus*, *Paracoccus*, and *Dietzia* [15,16,17,18].

Canthaxanthin production from bacteria has some limitations, such as low yield and safety concerns associated with pathogenic pigment-producing strains. Consequently, it is essential to select suitable microbial strains and optimize cultivation conditions to enhance pigment yield and ensure efficient production. Another factor that can significantly enhance production efficiency is minimizing the risk of contamination by undesirable microorganisms. Using a microbial strain with high tolerance to cultivation conditions that are unfavorable to general microbial contaminants is a promising approach. Strains belonging to *Paracoccus*, particularly *P. bogoriensis*, which is considered an extremophile because it is thermotolerant, halotolerant, and able to grow under alkaline conditions [19], are potential candidates for this purpose.

The unique structures of bacteria and their ability to produce functional biomolecules contribute significantly to their survival in extreme environments and stress tolerance [20,21]. Conversely, environmental stress, such as oxidative stress, can enhance the biosynthesis of certain biomolecules. For example, exposure to reactive oxygen species (ROS) can damage cellular components and trigger cellular defense mechanisms. As a primary defense, carotenoids, with their lipophilic properties, integrate into bacterial cell membranes and function as antioxidants, quenching singlet oxygen and scavenging free radicals. This mechanism prevents membrane lipid peroxidation, preserves membrane integrity, and regulates membrane fluidity [22].

Another stress factor affecting carotenoid production is pH. Due to the stress response mechanism, the optimum environmental factors for cell growth and maximum carotenoid accumulation may not be identical. For example, *Paracoccus carotinifaciens* had optimum cell growth and canthaxanthin production at pH 7.0 [17], whereas a salt-lake *P. bogoriensis* strain (BOG6) was reported to have an optimum pH for growth, astaxanthin, and canthaxanthin production of pH 9.5 [19]. Increased carotenoid production in response to pH stress has been observed in other microorganisms, such as the microalgae *Haematococcus lacustris* [23]. Similarly, Hegazy et al. [24] reported that the pH of whey medium influenced carotenoid production in *Micrococcus luteus*; carotenoid content increased with increasing pH from 5 to 7, remained constant at neutral pH values, and was inhibited at alkaline pH. However, the enhancement of carotenoid production by applied pH changes alone or in combination with other stress conditions in bacteria has been investigated in only a few studies, and it may vary by species and strains.

*Paracoccus* is a Gram-negative bacterium belonging to the class Alphaproteobacteria. Members of this genus are well known for their metabolic versatility and adaptability to a wide range of environments, including extreme ones. Many species are natural dwellers in extreme habitats such as marine environments, salt lakes, and hot springs [19,25,26]. Among the members of this genus, *P. bogoriensis* is one of the species recognized as an extremophile due to its characteristics that are linked to its ability to thrive in various extreme conditions. Certain strains are alkaliphilic, halotolerant, and thermotolerant. *P. bogoriensis* strains are known for their ability to produce carotenoid pigments. Together with their other extremophilic properties, these characteristics make them one of the most potentially useful microorganisms for biotechnological applications.

Therefore, this study aimed to address the limitations of natural canthaxanthin production from bacterial sources, focusing on a thermotolerant strain, *P. bogoriensis* PH1, as the subject of the study. This strain was retrieved from the Biobank of the Microbial Resource and Technology (MRT) Laboratory, Chiang Mai University, and had a history of producing bright orange pigments. It was of hot spring origin, with no history of being an opportunistic pathogen or causing infection in humans. This study identified potential strains, investigated key factors influencing pigment production, and developed a more efficient fermentation process that increases the yield while reducing the risk of microbial contamination. The study was performed in two phases: flask-scale to evaluate production variables, followed by optimization of parameters that affect growth and canthaxanthin production by the microorganism in an experimental-scale bioreactor to maximize pigment yields.

## 2. Materials and Methods

### 2.1. Strain Selection and Inoculum Preparation

*Paracoccus bogoriensis* PH1, a carotenoid-producing strain originally isolated from a hot spring at 40 °C by our group and obtained from the Biobank of the MRT Laboratory of Chiang Mai University, was used in this study. The 16S rRNA sequence (accession no. PX102350 (NCBI database)) indicated its identity as *Paracoccus bogoriensis*. Preliminary assessment using thin-layer chromatography indicated that this strain is a potential canthaxanthin producer. The activated culture was grown in Tryptic Soy Broth with 0.6% Yeast Extract or Tryptic Soy Yeast Extract Broth (TSYEB) (BD, Sparks, MD, USA) (pH 7.0) for 24 h at 37 °C. Following incubation, the bacterial suspension was adjusted to an OD_600_ of 0.2 and used as an inoculum for canthaxanthin production optimization experiments.

### 2.2. Determination of Pigment Content and Cell Dry Weight

Total pigments were determined according to the method described by Casella et al. [27]. Briefly, bacterial cells were collected after culturing under different conditions by centrifugation at 11,604× *g* for 15 min. The cell pellet from each sample was separated into two portions for pigment content and cell dry weight assessments and subsequently washed with sterile deionized (DI) water. To the first portion (cell pellet from 100 mL of broth culture with an OD_600_ of 0.5), 15 mL of acetone (RCI Labscan Limited, Bangkok, Thailand) was added. The tube was shaken until the cells became colorless, and the cell pellet was collected by centrifugation, as described above. The absorbance at 474 nm (strong absorption peak for canthaxanthin) was measured using a UV-visible spectrometer (BMG LABTECH, Ortenberg, Germany), and the pigment content was calculated from a linear standard curve of canthaxanthin, which was used as a representative carotenoid. The second portion of the cell pellet was dried in a hot air oven at 70 °C, and the dry cell weight was measured. All experiments were performed in triplicate.

### 2.3. Investigation of the Effects of pH and Temperature on Growth and Pigment Production in Flask Culture

The optimization of pigment production by *P. bogoriensis* PH1 was initially conducted at the flask scale using 100 mL of PH1 culture in TSYEB. A 1% inoculum (prepared as described in Section 2.1) of *P. bogoriensis* PH1 was introduced into 100 mL of TSYEB medium contained in a 250 mL Erlenmeyer flask and cultured under varied intrinsic and extrinsic factors (described below). All experiments were performed in triplicate.

pH: The PH1 strain was cultured at 37 °C for 72 h (stationary phase) in TSYEB with varying pH values (pH 6–12).

Temperature: The PH1 strain was cultured in TSYEB (pH 11) at 30, 35, 40, and 45 °C.

Another experiment was conducted at 40 °C using TSYEB with different initial pH values: pH 7 for 72 h (treatment 1), pH 11 for 72 h (treatment 2), and pH 7 for 72 h followed by pH 11 for an additional 24 h (pH 7»11) (treatment 3).

Nitrogen sources: The PH1 strain was cultured in the original TSYEB medium (1.7% tryptone (pancreatic digest of casein) and 0.3% soy peptone (papaic digest of soybean) as nitrogen sources). In comparison, it was also cultured in modified media, with tryptone and soy peptone replaced with inorganic and organic nitrogen compounds [28] at a 2% (*w*/*v*) concentration. The inorganic nitrogen sources included monosodium glutamate (MSG, C_5_H_8_NNaO_4_), ammonium chloride (AC, NH_4_Cl) (both supplied by HiMedia, Thane West, India), and ammonium sulfate (AS, (NH_4_)_2_SO_4_; RCI Labscan Limited, Bangkok, Thailand). The organic nitrogen sources included polypeptone (HiMedia), peptone, and yeast extract (the latter two were supplied by BD, Sparks, MD, USA). Additionally, the best nitrogen source was tested at 0.5, 1.0, 1.5, 2.0, and 2.5% to determine its optimum concentration.

Carbon sources: The PH1 strain was cultured in a modified medium (2.5% polypeptone, 0.5% NaCl, 0.25% K_2_HPO_4_, 0.25% glucose, 0.6% yeast extract (pH 7, adjusted to 11 after 72 h); and modified media having the carbon source (0.25% glucose) replaced with other carbon sources [28], including fructose, galactose, lactose, sorbitol, sucrose, and glycerol (the first four supplied by Kemaus, Cherrybrook, NSW, Australia; the latter two by Thermo Fisher Scientific, Geel, Belgium). The best-performing carbon source was further tested at 0.1, 0.5, 1.0, 2.0, 3.0, and 4.0% to determine the optimum concentration.

Shaking speed and agitation: Shaking speeds of 150, 200, and 250 rpm were tested after nutrient source optimization, with the culture grown in 100 mL of the optimum medium. Additionally, the effect of agitation on pigment synthesis was examined by comparing bacteria grown in standard Erlenmeyer flasks with those grown in baffled flasks at the optimum shaking speed. Volumetric mass transfer coefficients (k_L_a) for these culturing conditions were calculated using the Calculator Tool (https://www.presens.de, accessed on 6 December 2023).

After cultivation, the bacterial cells were harvested, and the dry cell weight and total pigment content were measured.

### 2.4. Study of Growth Characteristics of P. bogoriensis PH1 in Bioreactor

The optimum culturing conditions in flasks were adopted for the bioreactor. A newly formulated culture medium, designated as Polypeptone Sucrose Yeast Extract (PPSYE) medium (containing per liter: 25 g polypeptone, 5 g NaCl, 2.5 g K_2_HPO_4_, 10 g sucrose, and 6 g yeast extract), was used. Fermentation (1 L volume) was performed in a 2 L bioreactor (MDFT-N-1 L, B.E. Marubishi, Pathum Thani, Thailand). A 10% (*v*/*v*) *P. bogoriensis* PH1 inoculum (OD_600_ of 1.0) was added to one liter of sterilized PPSYE medium in the bioreactor (pH 7.0) and incubated at 40 °C, with an agitation rate of 250 rpm, and 2 vvm aeration. The dissolved oxygen (DO) level in the culture medium was measured every 12 h, together with OD_600_ and viable cell count using the drop plating method. The pH-shift strategy was applied, with the pH of the culture medium adjusted to pH 11 using NaOH after 36 h of incubation (the culture reached the stationary phase, with an OD_600_ of 10^−1^ culture of 1.1–1.2 and a number of microorganisms of 9.4–9.5 log CFU/mL).

Bacterial cultures were sampled every 12 h for a total of 36 h after the pH shift, and the cell dry weight and total pigment content were determined as described in Section 2.2.

### 2.5. Carotenoids Extraction

Carotenoids were extracted from the cells harvested from the culture grown in the bioreactor using a method modified from Rodriguez-Amaya [2], as follows. Cells were harvested by centrifugation at 11,604× *g* for 15 min at 4 °C. One gram of wet cell biomass was mixed with 20 mL acetone. The cells were ground using a mortar and pestle and sonicated at room temperature for 20 min. The extract was filtered through anhydrous sodium sulfate (Fisher Scientific UK, Loughborough, UK) into a 100 mL separatory funnel and eluted with petroleum ether (RCI Labscan Limited, Bangkok, Thailand) until the sodium sulfate became colorless. Subsequently, 15–20 mL of distilled water was added, and the funnel was gently shaken three times for 5 s each time before removing the aqueous phase. The organic layer was filtered again through sodium sulfate into a 100 mL evaporation flask, and the sodium sulfate was rinsed with petroleum ether until it became colorless. The extract was dried using a rotary evaporator (Tokyo Rikakikai Co., Ltd., Tokyo, Japan) and redissolved in 10 mL of acetone. For saponification, 1 g of Ambersep^®^ 900 OH resin (strongly basic hydroxide form; Supelco, Sigma-Aldrich Chimie S.A.R.L., St. Quentin Fallavier, France) was added and stirred for 30 min. Water was removed again, and the crude extract was dissolved in petroleum ether, flushed with nitrogen, and stored at −20 °C in the dark.

### 2.6. Determination of Wavelength Maxima (λ_max_) and Total Carotenoid Content (TCC)

The dried carotenoid extract of *P. bogoriensis* PH1 was dissolved in petroleum ether, and the absorbance in the 200–800 nm range was measured using a UV–visible spectrophotometer with the ASpect UV program (Specord 210 Plus, Analytik Jena, Jena, Germany) to determine λ_max_ and TCC. Carotenoid standards, including astaxanthin, lutein, zeaxanthin, canthaxanthin, β-cryptoxanthin, and β-carotene (all from Dr. Ehrenstorfer, Augsburg, Germany), were scanned under identical conditions. The λ_max_ values of the extracts were compared with those of the standards. The absorbance at λ_max_ was used for TCC calculation according to the modified method described by De Carvalho et al. [29].

### 2.7. HPLC Analysis

Carotenoid pigments produced by the PH1 strain obtained from flask and bioreactor cultivation were identified using HPLC, according to the method described by Gupta et al. [30] with modifications. A solvent mixture of methyl tert-butyl ether (MTBE) and methanol (1:1) (HPLC grade; RCI Labscan Limited, Bangkok, Thailand) was used to dissolve the carotenoid extracts, which were filtered through a 0.22 µm nylon membrane(ALWSCI Technologies, Zhejiang, China) before injection into the HPLC system. The carotenoid standards used in this study were astaxanthin, lutein, zeaxanthin, canthaxanthin, β-carotene, and β-cryptoxanthin. A SpectraSYSTEM UV2000 system (Thermo Fisher Scientific, Waltham, MA, USA) was used for the HPLC analysis. A YMC carotenoid column, a C30 bonded silica-based reverse-phase column (5 µm, 250 mm × 4.6 mm) (YMC Co., Kyoto, Japan), was used. A 20 µL aliquot of each sample was injected into the HPLC system. The mobile phase consisted of a gradient system with solvents A (methanol/MTBE/water = 81:15:4) and B (methanol/MTBE = 10:90). Gradient elution was performed using 1% B from 0 to 39.0 min, 56–100% B from 39.0 to 39.1 min, and 100–1% B from 39.1 to 45.0 min, at a flow rate of 1 mL/min. The retention times of all carotenoid compounds were determined at 480 nm. Additionally, individual carotenoid standards were quantified using HPLC, and a calibration curve for each carotenoid, correlating the peak area at a specific retention time with the concentration, was plotted. This calibration curve was used to determine the amount of each carotenoid present in the sample.

### 2.8. Purification of Canthaxanthin by Thin-Layer Chromatography (TLC)

Canthaxanthin produced by *P. bogoriensis* PH1 under optimum bioreactor conditions was purified using the TLC method based on the procedure described by Lorquin et al. [31], with modifications. Briefly, the carotenoid extract was dissolved in dichloromethane (RCI Labscan Limited, Bangkok, Thailand) and applied onto a TLC plate (silica gel 60 GF254, 20 × 20 cm; Merck KGaA, Darmstadt, Germany). The plate was developed in a chamber saturated with a dichloromethane/ethyl acetate (99:1) mixture (RCI Labscan Limited, Bangkok, Thailand) as the mobile phase. The band corresponding to canthaxanthin was gently scraped from the TLC plate and transferred to a 250 mL Erlenmeyer flask containing 100 mL of 99.8% dichloromethane. The silica gel was then sonicated to elute the carotenoids until it became colorless. The eluted solutions were filtered through a layer of anhydrous sodium sulfate placed on filter paper in an evaporating flask. Sodium sulfate was subsequently rinsed with additional dichloromethane to recover any remaining compounds. The solution was concentrated by evaporation to obtain a dry state. The dried canthaxanthin was redissolved in a minimum volume of fresh dichloromethane and stored under a nitrogen atmosphere at –20 °C in the dark until further analysis was performed.

### 2.9. LC-MS/MS Analysis

Purified canthaxanthin was confirmed using LC-MS/MS analysis based on the method described by Balasubramaniam et al. [32] and Maoka [33], with modifications. An Agilent 6545 LC/QTOF system in conjunction with a 1260 Infinity II LC system (Agilent Technology, Inc., Santa Clara, CA, USA) was used with an Agilent Poroshell 120 EC-C18 column (2.7 µm, 4.6 × 100 mm) operated at 35 °C. The samples were dissolved in 99.9% methanol (HPLC grade; Honeywell, Charlotte, NC, USA) and filtered through a 0.22 µm nylon membrane filter prior to analysis. A 5 μL sample was then injected. Solvent mixtures A (water with 0.1% formic acid (HPLC grade; Merck KGaA, Darmstadt, Germany)) and B (acetonitrile (Honeywell, Charlotte, NC, USA) with 0.1% formic acid) were employed in a gradient mode as follows: an initial run from 0 to 2 min with 5% of solvent B, followed by an increase from 2 to 20 min with the solvent B increasing from 5% to 99%, a hold from 20 to 25 min with 99% of solvent B, then a run from 25 to 26 min with the solvent B decreasing from 99% to 55%, and a final run from 26 to 30 min with 99% of solvent B, at a flow rate of 0.5 mL/min. The resolving power was set to 140,000 FWHM. The acquisition mode was targeted (MS/MS), focusing on a target *m*/*z* of 565.4018. The MS and MS/MS ranges were set between 100 and 800 *m*/*z*. Collision energies of 10 V were selected for analysis. Electrospray ionization (ESI) was performed in positive mode. The temperature of the drying gas was set to 320 °C at a flow rate of 8 L/min, and the nebulizer was set to 35 psi. The sheath gas temperature was set to 350 °C with a flow rate of 11 L/min. The capillary voltage (Vcap) and nozzle were set to 3500 and 1000 V, respectively. The TOF analyzer was set with a fragmentor voltage of 175 V, skimmer voltage of 65 V, and octupole RF voltage of 750 V. The METLIN database, which combines information from KEGG, HMDB, ChEBI, and BioCyc, was used for analysis.

### 2.10. Determination of Antioxidant Activity of Purified Canthaxanthin Using the DPPH and ABTS Assays

Purified canthaxanthin was initially dissolved in 50 μL of DMSO and subsequently diluted with 99.9% methanol (RCI Labscan Limited, Bangkok, Thailand) to obtain various concentrations ranging from 0.025 to 0.4 mg/mL. This preparation was used in the DPPH and ABTS assays.

The DPPH assay was performed as described by Naranjo-Durán et al. [34]. Each of the positive control solutions (ascorbic acid and Trolox, prepared at 0.1–5.0 μg/mL) and 50 μL canthaxanthin solution were mixed with 50 μL of DPPH (Sigma-Aldrich, Saint Louis, MO, USA) solution in a 96-well plate and incubated in the dark at room temperature for 30 min. After incubation, the absorbance was measured at 517 nm using a microplate reader (Multiskan GO, Thermo Scientific, Vantaa, Finland) with SkanIt Software 3.2. The assays were performed in triplicate.

The ABTS assay was modified from the method described by Chang et al. [35]. Each sample (50 μL) and the positive control solution (Trolox, prepared in the range of 0.2–3.13 μg/mL) were mixed with 150 μL of ABTS (Sigma-Aldrich, Saint Louis, MO, USA) in a 96-well plate and incubated in the dark at room temperature for 5 min. After incubation, the absorbance of the reaction mixture was measured at 734 nm using a microplate reader. The assays were performed in triplicate. A calibration curve of ABTS scavenging activity versus concentration was used to determine the IC_50_.

### 2.11. Statistic Analysis

Statistical analyses were performed using SPSS Statistics version 19 (IBM Corp., Armonk, NY, USA). One-way ANOVA was employed to compare the means across multiple groups, with Tukey’s post-hoc test applied for pairwise comparisons (*p* < 0.05). A paired-samples *t*-test was employed to compare the mean values of individual carotenoids between the pre-optimization conditions in flasks and the optimized conditions in the bioreactor (*p* < 0.05 and *p* < 0.01).

## 3. Results

### 3.1. Effects of Intrinsic and Extrinsic Factors on Pigment Production by P. bogoriensis PH1 in Flask Culture

**Figure 1:**
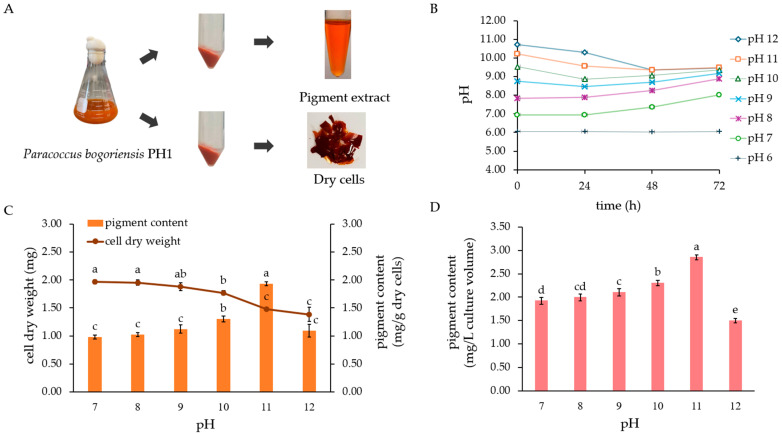
Cell biomass and pigment production by *P. bogoriensis* PH1 in flask culture in Tryptic Soy Yeast Extract Broth (TSYEB) with various initial pH values. (**A**) Separation of culture for determination of cell dry weight and pigment production by strain PH1, (**B**) changes in the pH of TSYEB (initial pH 6–12) during cultivation for 72 h, (**C**) cell dry weight and pigment content per gram dry cells obtained as a result of culturing under different pH values, and (**D**) pigment content per liter, calculated from the canthaxanthin standard curve. Error bars represent SD (*n* = 3). Different letters represent statistically significant differences (Tukey’s HSD test, *p* < 0.05).

*P. bogoriensis* PH1 was initially assessed for growth and pigment production under various pH conditions, with initial pH values ranging from 6 to 12. It demonstrated growth in TSYEB medium with an initial pH of 7 to 12, as indicated by an increase in OD_600_ from 0 to 72 h, but no growth at pH 6 (the OD_600_ remained unchanged) (Supplementary Figure S1A). The cell dry weight and pigment content of the PH1 culture grown under different initial pH values are shown in Figure 1. The TSYEB culture grown at an initial pH of 7 and 8 resulted in the highest cell dry weight of 1.97± 0.03 mg/mL. As the medium alkalinity increased, the cell dry weight gradually decreased, whereas the pigment content significantly increased up to pH 11. At pH 11, the strain produced the highest pigment content (1.93 ± 0.04 mg/g dry cells and 2.86 ± 0.05 mg/L culture volume) (Figure 1C,D). Therefore, the TSYEB medium with an initial pH of 11 was selected for subsequent studies, as it was the optimum pH for pigment production by the PH1 strain. Because the cell dry weight and pigment content (mg/g dry cells) under different pH levels were not proportionally correlated, the pigment content per culture volume was mainly used in subsequent pigment optimization experiments.

**Figure 2:**
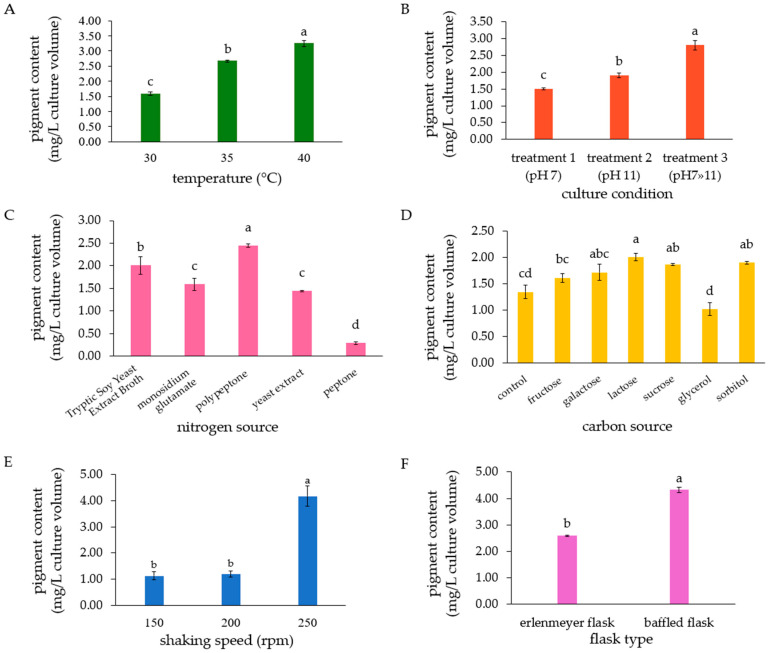
Effects of intrinsic and extrinsic factors on pigment production by *P. bogoriensis* PH1: (**A**) temperature (cultured in TSYEB, pH 11), (**B**) pH (cultured in TSYEB at 40 °C), (**C**) nitrogen sources (cultured in Yeast Extract Broth with 2% (*w*/*v*) of each nitrogen source added in place of tryptone and soy peptone under the pH-shift conditions (pH 7»11)), (**D**) carbon sources (cultured in medium having 2.5% polypeptone as nitrogen source and 0.25% (*w*/*v*) of each carbon source added in place of glucose), (**E**) shaking speeds (cultured in Polypeptone Sucrose Yeast Extract (PPSYE) Broth (pH 7»11)), (**F**) flask types (cultured in PPSYEB (pH 7»11)). The pigment content was calculated using a canthaxanthin standard curve. Data are presented as means ± SD. Different letters indicate statistically significant differences (Tukey’s HSD test; *p* < 0.05).

For temperature optimization, *P. bogoriensis* PH1 was cultured in TSYEB medium (with an initial pH of 11) at various temperatures (30, 35, 40, and 45 °C). The results showed that the strain could grow at temperatures between 30 and 40 °C, as observed from the increasing OD_600_ values during the 72 h period, but not at 45 °C (Supplementary Figure S1B). The highest cell dry weight was obtained at 35 °C (1.54 ± 0.06 mg/mL), followed by 40 °C (1.32 ± 0.04 mg/mL). This suggests that *P. bogoriensis* PH1 is a mildly thermotolerant bacterium. However, the highest pigment content was achieved at 40 °C (3.25 ± 0.10 mg/L) (Figure 2A).

To maximize pigment production after achieving a high cell density, pigment production was induced using a pH shift strategy. The PH1 strain was grown for 72 h at 40 °C in TSYEB with an initial pH of 7 (treatment 1), TSYEB with an initial pH of 11 (treatment 2), and TSYEB with an initial pH of 7 that was later adjusted to pH 11 after 72 h and further incubated for 24 h (treatment 3). At 72 h, the culture was in the stationary phase, with viable cell counts of approximately 9 log CFU/mL. The highest pigment content (2.80 ± 0.14 mg/L) was achieved with treatment 3 (Figure 2B).

Optimization involving nutrient factors was subsequently performed using the cultivation conditions of treatment 3. The tested nitrogen sources included inorganic nitrogen sources (MSG, AC, and AS) and organic nitrogen sources (polypeptone, peptone, and yeast extract). Each nitrogen source was added at 2% (*w*/*v*), replacing the original nitrogen sources (1.7% tryptone and 0.3% soy peptone) in TSYEB medium. Among the inorganic nitrogen sources tested, only MSG supported both growth and pigment production by the PH1 strain, yielding a pigment content of 1.58 ± 0.13 mg/L. As for organic nitrogen sources, the bacterium grew and produced pigments in media containing all three nitrogen sources tested. The highest pigment yield of 2.44 ± 0.04 mg/L was achieved with the medium containing polypeptone, which was significantly higher than that achieved with TSYEB (2.01 ± 0.19 mg/L) (Figure 2C). The effects of polypeptone on growth and pigment production were then tested at concentrations of 0.5, 1.0, 1.5, 2.0, and 2.5%. The pH1 strain was able to grow and produce pigments at all tested concentrations, but the highest pigment content was obtained at 2.5%, which was significantly different from that obtained at other concentrations (Supplementary Figure S2). Therefore, 2.5% polypeptone was used to replace tryptone and soy peptone in subsequent experiments.

Next, the optimum carbon source for total pigment production by *P. bogoriensis* PH1 was determined. The selected carbon sources included monosaccharides (fructose and galactose), disaccharides (lactose and sucrose), and sugar alcohols (sorbitol and glycerol). These were used to replace the original 0.25% glucose in the medium. The bacterium was cultured in the medium (pH 7) with 2.5% polypeptone as the nitrogen source at 40 °C. After 72 h, pH was adjusted to 11, and incubation continued for another 24 h. As a result, the bacterium produced the pigment in media containing lactose, sucrose, sorbitol, and galactose at concentrations of 2.01 ± 0.07, 1.87 ± 0.02, 1.90 ± 0.03, and 1.72 ± 0.15 mg/L, respectively, which are not significantly different (*p* > 0.05). Nevertheless, sucrose was selected because it is easily available and more cost-effective than other sugars that yielded similar pigment contents. Sucrose was then incorporated in the medium in the range of 0.1 to 4%. The results showed that 1 and 2% (*w*/*v*) sucrose were the optimum concentrations for pigment production (Supplementary Figure S3). A sucrose concentration of 1% was selected for subsequent experiments.

As a result of nutrient optimization, a new culture medium, designated as Polypeptone Sucrose Yeast Extract (PPSYE) medium, was formulated. The composition per liter was as follows: 25 g polypeptone, 5 g NaCl, 2.5 g K_2_HPO_4_, 10 g sucrose, and 6 g yeast extract.

Thereafter, the effects of shaking speed on pigment production were investigated at 150, 200, and 250 rpm. A shaking speed of 250 rpm resulted in the highest pigment content (4.18 ± 0.39 mg/L), which was significantly higher than that obtained at lower shaking speeds (Figure 2E) and was selected for further experiments.

The results on shaking speed and pigment production suggest that *P. bogoriensis* PH1 requires high-aeration conditions for pigment production. Therefore, a further experiment was conducted to compare the effects of agitation on pigment synthesis using Erlenmeyer flasks and baffled flasks at a shaking speed of 250 rpm. Culturing the bacteria in baffled flasks (k_L_a = 334 h^−1^) resulted in a pigment concentration of 4.32 ± 0.09 mg/L, significantly higher (*p* < 0.05) than that obtained from culturing in Erlenmeyer flasks (k_L_a of 41 h^−1^), which was 2.59 ± 0.03 mg/L (Figure 2F). These results confirmed that strain PH1 could grow and produce high pigment yields under high aeration, and the optimum conditions in this experiment were considered when designing the aeration and agitation rates for the bioreactor-scale experiment.

### 3.2. Growth Characteristics and Pigment Production by P. bogoriensis PH1 in Bioreactor

**Figure 3:**
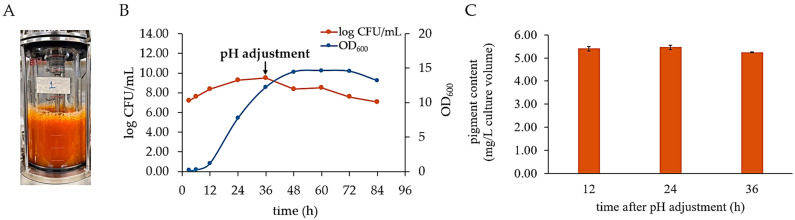
Pigment production by *P. bogoriensis* PH1. (**A**) Culture in a lab-scale bioreactor, (**B**) growth characteristics of the strain in PPSYE broth in the bioreactor, and (**C**) pigment contents (data are presented as means ± SD, *n* = 3) from bacterial cells harvested at different time points following pH adjustment, calculated from a canthaxanthin standard curve. No significant differences were observed (Tukey’s HSD test; *p* < 0.01).

Fermentation was performed in a 2 L bioreactor with a working volume of 1 L (Figure 3A). The temperature was maintained at 40 °C, with an aeration rate of 2 vvm (the maximum rate) and a speed of 250 rpm (having a k_L_a value of 552 h^−1^).

The results shown in Figure 3B illustrate the growth characteristics of *P. bogoriensis* PH1 in the bioreactor. The viable count was at its peak at 36 h, a point in the stationary phase at which the viable count reached approximately 9 log CFU/mL. Therefore, this time point was selected for pH adjustment in the present study. Additionally, monitoring of the dissolved oxygen levels revealed complete oxygen depletion at 24 h.

The cell harvesting time for pigment extraction was tested at 12, 24, and 36 h after pH adjustment. As shown in Figure 3C, the highest pigment contents derived from the cells harvested 12, 24, and 36 h after pH adjustment were not significantly different. Therefore, cells were harvested 12 h after pH adjustment, as this yielded a high pigment content while reducing the overall production time. Furthermore, cultivation of strain PH1 in a bioreactor resulted in a productivity of 5.40 ± 0.10 mg/L of culture volume, representing 1.25- and 2.79-fold increases compared to those obtained under optimum conditions in baffled flasks and pre-optimization conditions in Erlenmeyer flasks, respectively.

### 3.3. Carotenoid Production by P. bogoriensis PH1 in Bioreactor

**Figure 4:**
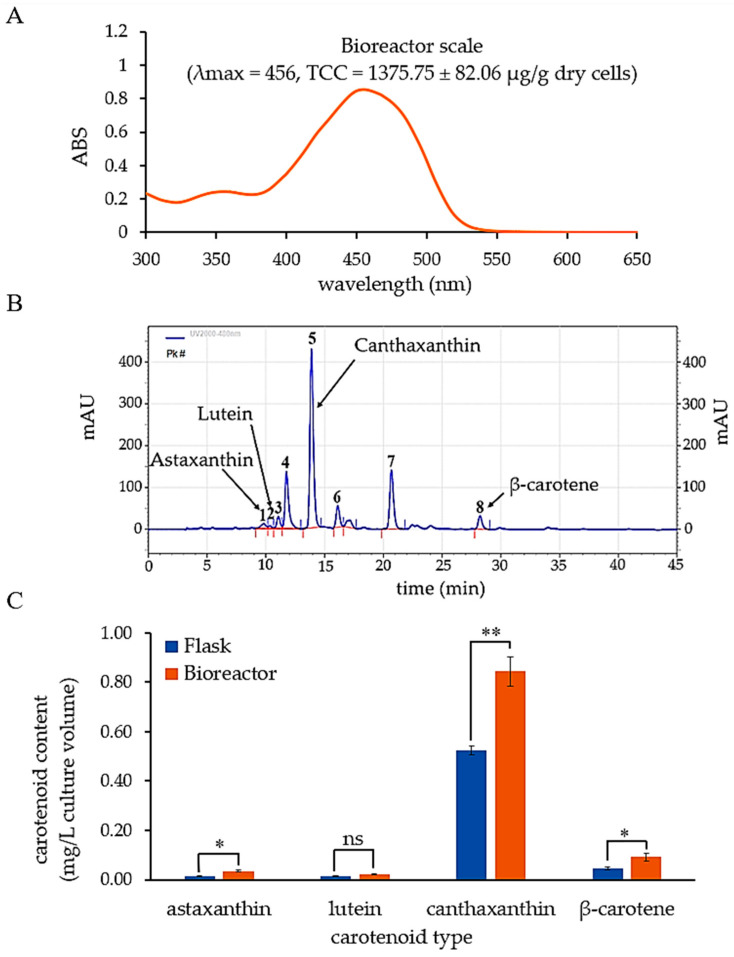
(**A**) Absorbance spectrum and λ_max_ of carotenoid extract from *P. bogoriensis* PH1 cultivated in a bioreactor, (**B**) HPLC chromatogram of the carotenoid extract, showing canthaxanthin as a major carotenoid produced by the PH1 strain, and (**C**) increase in carotenoid content after optimization in the bioreactor compared to pre-optimization in the flask. The carotenoid content was quantified using individual carotenoid standard curves generated by HPLC analysis. The numbers in the HPLC chromatogram indicate the peaks representing single compounds, with the identified carotenoid compounds indicated by the arrows. Data are presented as means ± SD. Statistically significant differences (Tukey’s HSD, *p* < 0.05) are shown by asterisks; *: *p* < 0.05, **: *p* < 0.01, ns: not significantly different (paired-samples *t*-test).

*P. bogoriensis* PH1 was cultivated under optimum conditions in a bioreactor, and the cells were harvested for pigment extraction. Scanning pigment absorbance within the wavelength range of 400–500 nm revealed a λ_max_ of 456 nm (Figure 4A). The mean TCC obtained under optimum conditions in the bioreactor was 1375 ± 82.06 μg/g dry cells, representing an approximately 3.12-fold increase compared to the total carotenoid content obtained from the flask culture prior to optimization. Therefore, these optimized conditions effectively enhanced total carotenoid biosynthesis by *P. bogoriensis* PH1 at the bioreactor-scale.

The pigment extract was further analyzed for specific carotenoid types using HPLC. The HPLC chromatogram of the carotenoid extract (Figure 4B) shows that *P. bogoriensis* PH1 produces several carotenoids, including astaxanthin, lutein, canthaxanthin, and β-carotene. The concentrations of astaxanthin, lutein, canthaxanthin, and β-carotene were 0.04, 0.02, 0.84, and 0.09 mg/L, respectively, and were higher in cultures grown in the bioreactor under the optimized conditions than in the pre-optimized culture, with increases of 2.70-, 1.53-, 1.61-, and 2.05-fold, respectively (Figure 4C).

### 3.4. HPLC and LC-MS/MS Analysis of Purified Canthaxanthin

**Figure 5:**
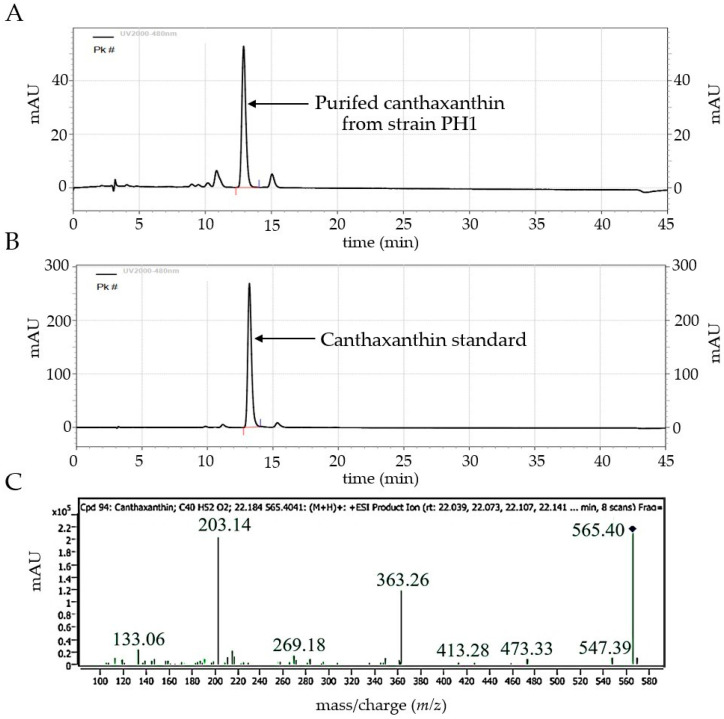
Confirmation of purified canthaxanthin produced by *P. bogoriensis* PH1 using HPLC and LC-MS/MS analysis: (**A**) HPLC chromatogram of purified canthaxanthin (The peaks (Pk#) were detected at 480 nm using UV2000 detector), (**B**) HPLC chromatogram of canthaxanthin standard, and (**C**) LC-MS/MS fragment ions of canthaxanthin (with the *m*/*z* values of specific fragment ions given with the peaks).

Canthaxanthin, purified using TLC, was confirmed using HPLC and LC-MS/MS. The HPLC chromatogram of the purified canthaxanthin exhibited a retention time (RT) of 12.902 min, closely matching that of the standard canthaxanthin (13.207 min) (Figure 5A,B). The purified canthaxanthin was further confirmed using LC-MS/MS analysis. The LC-MS/MS results were compared with those in the METLIN database, which integrates data from KEGG, HMDB, ChEBI, and BioCyc. LC-MS/MS analysis of the purified canthaxanthin revealed a retention time of 22.184 min and a molecular mass of 564.3970. The [M + H]^+^ molecular ion was observed at an *m*/*z* ratio of 565.4041, corresponding to protonated canthaxanthin, with a peak at an *m*/*z* ratio of 565.4029. Additional fragment ions generated by electrospray ionization (ESI) at a collision energy of 10 V were observed at *m*/*z* ratios of approximately 547, 473, 413, 363, 269, 203, and 133 (Figure 5C). These fragmentation patterns matched those of canthaxanthin (C_40_H_52_O_2_), with a target score of 98.52%.

### 3.5. DPPH and ABTS Radical Scavenging Activity of Canthaxanthin Produced by P. bogoriensis PH1

The antioxidant activities of purified canthaxanthin from *P. bogoriensis* PH1 were evaluated using DPPH and ABTS assays and compared with those of standard canthaxanthin, ascorbic acid, and Trolox. At the highest concentration (0.4 mg/mL), standard canthaxanthin and purified canthaxanthin exhibited comparable DPPH radical-scavenging activities (11.84 ± 3.89% and 11.23 ± 0.25%, respectively), with IC_50_ values greater than 0.4 mg/mL. However, ascorbic acid and Trolox exhibited significantly higher activity, with IC_50_ values of 1.97 μg/mL and 2.64 μg/mL, respectively (Supplementary Table S1).

**Figure 6:**
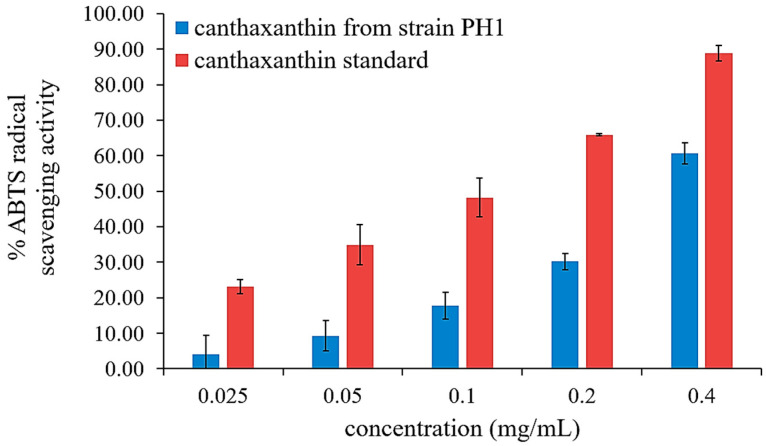
ABTS radical-scavenging activity of canthaxanthin (purified from extract) produced by *P. bogoriensis* PH1 compared with canthaxanthin standard (prepared from dry powder), *n* = 3. The error bars show ± SD.

In the ABTS assay, at a concentration of 0.4 mg/mL, the ABTS scavenging activity was 88.84 ± 2.21% for the standard canthaxanthin and 60.66 ± 2.94% for the purified canthaxanthin from *P. bogoriensis* PH1 (Figure 6). The IC_50_ values of standard canthaxanthin and purified canthaxanthin extracted from the cells of strain PH1 were 0.15 ± 2.22 mg/mL (150 μg/mL) and 0.33 ± 2.94 mg/mL (330 μg/mL), respectively, which were higher than that of Trolox (1.75 μg/mL).

## 4. Discussion

Carotenoid pigment production in *P. bogoriensis* PH1 was closely associated with environmental stress conditions. Interestingly, regardless of whether the initial pH of the medium was neutral or highly alkaline (pH 12), the final pH consistently converged to approximately 9, indicating that the strain actively modulates the pH of the medium to establish a favorable environment. The pH change in the culture medium during bacterial growth depends on its composition, such as carbon or nitrogen sources that are metabolized into acidic or alkaline compounds, causing the medium to shift towards acidity or alkalinity. The TSYEB medium is rich in proteins and amino acids and contains glucose as a carbohydrate source. With initial pH values of 7 and 8, the strain may first ferment the carbohydrates, causing a drop in pH. Once carbohydrates are depleted, the bacteria catabolize proteins and amino acids, which then raises the pH and makes the media alkaline. Moreover, the ability to adjust the pH to a level suitable for growth is regulated by genetic mechanisms or feedback loops [36]. This can also cause the final pH of the culture medium to converge to approximately 9.

While optimum growth occurred under mesophilic and neutral conditions (35 °C, pH 7), carotenoid production was markedly enhanced under stress conditions, particularly at an elevated temperature and an alkaline pH (40 °C, pH 11). This uncoupling of growth and pigment synthesis, also observed in previous studies [37,38], supports the notion that the production of carotenoid, especially cantaxanthin, in this strain functions as a stress response mechanism, with pH stress playing a central role in driving pigment synthesis, independently of biomass accumulation. Hu et al. [39] found that pH 6 was optimum for the growth of yeast *Xanthophyllomyces dendrorhous*, whereas pH 4 was optimum for its astaxanthin production. The pH-shift process developed during the cultivation of this yeast strain increased the astaxanthin concentration in batch fermentation by 24.1% compared to that in constant pH fermentation. Similarly, Dias et al. [40] found that the two-stage pH control method significantly enhanced the carotenoid yield of *Rhodotorula toruloides* compared with a single pH condition. The culture medium was first adjusted to pH 4 to promote rapid cell growth and was subsequently maintained at pH 5 to optimize carotenoid production. These observations prompted the development of a pH shift strategy, or two-stage pH control, during fermentation to enhance carotenoid pigment production.

The increased carotenoid production observed under pH stress can be attributed to the cellular response of microorganisms exposed to highly acidic or alkaline conditions. Such environments induce oxidative stress via the intracellular generation of reactive oxygen species (ROS). To mitigate these effects, some bacterial cells synthesize carotenoids, which function as ROS scavengers, thereby contributing to cellular survival [41]. In addition, carotenoids are known to modulate the biophysical properties of cellular membranes, supporting the notion that the observed phenotypes may result from alterations in membrane organization under stress conditions [42].

In addition, *P. bogoriensis* PH1 could grow at 35 and 40 °C. Nevertheless, the highest pigment yield was obtained from the culture grown in TSYEB with an initial pH of 11 and an incubation temperature of 40 °C. This indicates that temperature stress may be another key factor that promotes pigment production. This aligns with the study of Cruz-Angeles et al. [43], in which low temperature resulted in slower growth of *Halorubrum salinarum* RHB-CT but stimulated the production of carotenoids, including haloxanthin, bacterioruberin (BRB), and monoanhydro-bacterioruberin (MABR). Our experiment using the pH shift strategy allowed for high biomass accumulation prior to the introduction of alkaline stress, which significantly enhanced pigment production. This strategy was observed in another study [44].

Regarding nitrogen sources, organic nitrogen sources can provide not only nitrogen but also other essential nutrients, such as amino acids, vitamins, and growth factors, which are crucial for the metabolism of microorganisms. A high C/N ratio resulting from the slow release of nitrogen from an organic source may also contribute to the induction of pigment production [45]. From our experiment, although MSG best supported pigment production among the inorganic nitrogen sources tested, the bacterium exhibited better growth and pigment production when using organic nitrogen sources, especially polypeptone. These results suggest that MSG can serve as a simple nitrogen source that can be converted into glutamate and directly fed into metabolic pathways, such as the TCA cycle, leading to rapid nitrogen assimilation and enhanced bacterial growth [46]. 

Polypeptone is a mixture of enzymatic digests of casein and animal tissues. It supports the growth of a wide range of bacteria by supplying a broad spectrum of peptides and amino acids. It is slowly assimilated by bacteria because it consists of larger and more complex peptides, which can result in temporary nitrogen limitation that induces carotenoid production [47]. However, the optimum nitrogen source is specific to each microorganism. For example, yeast extract enhanced carotenoid production to a maximum amount in the UV-resistant strain of *Dietzia maris* [48]. Peptone with yeast extract was the optimum nitrogen source for canthaxanthin production by *Dietzia natronolimnaea* HS-1 [49]. Moreover, carotenoid-producing cyanobacteria are known for their nitrogen fixation, and some bacterial species in the genus *Paracoccus* are known to have denitrification potential, which is important for nitrogen cycling [50,51] and can support sustainable microbial-based nitrogen strategies in biotechnological fermentation.

As for carbon sources, sucrose was identified as the optimum carbon source for maximizing pigment yield in *P. bogoriensis* PH1. Its concentrations also affected the pigment yield. These findings align with those of Jeong et al. [52], who reported that sucrose was the optimum carbon source for the production of the carotenoid deinoxanthin, in the extremophilic bacterium *Deinococcus radiodurans* when compared with glucose and fructose. Naik and Gupte [53] reported that complex carbon sources (such as soluble starch and lactose) are more favorable than simple ones (such as glucose and fructose) for pigment production by *Paracoccus marcusii* RSPO1. Similarly, Afra et al. [54] observed a significant increase in pigment production by *Arthrobacter* sp. when cultured complex carbon sources, such as maltose.

In our study, a newly modified medium, Polypeptone Sucrose Yeast Extract (PPSYE), was formulated as a result of nutrient optimization for high-pigment production by *P. bogoriensis* PH1. This modified medium had the original nitrogen sources (tryptone and soy peptone) and carbon source (glucose) of TSYEB replaced with polypeptone and sucrose, respectively. Moreover, pigment yield was enhanced by culturing strain PH1 in PPSYE broth at high aeration rates.

The conditions for pigment production by *P. bogoriensis* PH1 optimized at the flask scale were adopted for the bioreactor-scale production. The pH-shift strategy in combination with an above-optimum temperature and other optimized parameters, including aeration, time for pH adjustment, and harvesting time after pH adjustment, provided the highest pigment yield while reducing the overall process duration.

The optimization employed a natural approach to enhance the total carotenoid production. Subsequent HPLC analysis indicated that the predominant carotenoid pigment produced by the strain was canthaxanthin, which is in agreement with the bright orange color observed by eye and the analysis by TLC. The minor carotenoids included astaxanthin, lutein, and β-carotene. These findings suggest that *P. bogoriensis* PH1 has strong potential as a source of canthaxanthin and possibly other valuable carotenoids.

Carotenoid production depends primarily on the strain capacity, and the culturing conditions can determine the type(s) of specific carotenoids produced and enhance their yields. Naik and Gupte [53] reported that *Paracoccus marcusii* RSPO1 produced a high carotenoid yield of 7240 ± 41 μg/L under optimum conditions (cultured in medium containing 2% starch with 0.5% malt extract, pH 7, at 37 °C with shaking at 100 rpm). Another study found that an extreme halophile *Haloferax alexandrines* strain, TMT, cultured under optimum conditions (100 mL of CM Broth containing 25% NaCl at pH 7.2 in a 500 mL Erlenmeyer flask with a shaking speed of 240 rpm, incubated at 37 °C for 6 days), produced 2.06 mg of total carotenoids per gram of dry cells. The carotenoids included β-carotene (2.94%), 3-hydroxy-echinenone, bacterioruberins (63.17%), and canthaxanthin (33.88%). No growth or pigment production was observed above pH 7.5, and both growth and pigment production declined at temperatures above and below 37 °C [55].

From the above reports, it can be seen that the canthaxanthin yield obtained from *P. bogoriensis* PH1 under optimum conditions was in the expected range of pigments produced by microorganisms. For *P. bogoriensis* PH1, batch culture in a laboratory-scale bioreactor resulted in a significant increase in multiple carotenoids from the pre-optimized conditions: a 2.70-fold increase in astaxanthin, a 1.53-fold increase in lutein, a 1.61-fold increase in canthaxanthin, and a 2.05-fold increase in β-carotene contents. The increases were not equal for all carotenoid types, but the yield might be further increased by further optimization, such as using a fed-batch culture or a specific direction of metabolic pathways. Since pH is one of the key factors determining carotenoid production, other pH control strategies can also be used, which may be applied in combination with other factors, as demonstrated in this study. For example, Han et al. [23] reported that applying pH shock (pH = 4.5 for 60 s) enhanced astaxanthin biosynthesis in *Haematococcus lacustris*, resulting in a 39 ± 6.92% increase in astaxanthin accumulation. When combined with high irradiance or carbon source supplementation, the astaxanthin yield increased further to 65 ± 0.541% and 105 ± 6.66%, respectively.

In terms of raw material cost, polypeptone represents a significant expense, and maintaining aerated, controlled conditions requires additional energy. However, the reduced risk of contamination and the elimination of antibiotics and sterilization steps may help offset some of these expenses. A comprehensive techno-economic assessment is essential for fully evaluating the potential for industrial applications.

The purified compound was conclusively identified as canthaxanthin based on its LC-MS/MS profile. The [M+H]^+^ ion showed a low-intensity neutral loss of toluene (*m*/*z* 473) [56] and produced a fragment ion at *m*/*z* 203.1, indicative of carotenoids containing a keto group as the sole substituent on the β-ring conjugated to the polyene chain [57]. Furthermore, Maoka [33] reported that canthaxanthin typically produces product ions at *m*/*z* 473 ([M+H–92]^+^), 459 ([M+H–106]^+^), 427, 361, 347, 215, 203, and 133, which is consistent with the LC-MS/MS data obtained from the extract of *P. bogoriensis* PH1 cells.

Purified canthaxanthin from *P. bogoriensis* PH1 culture grown in a bioreactor under optimum conditions exhibited antioxidant activity, especially in the ABTS assay, demonstrating its electron-donating capabilities to neutralize positively charged free radicals, as evidenced by the ABTS assay results. Gharibzahedi et al. [58] investigated the antioxidant activity of canthaxanthin synthesized by *D. natronolimnaea* HS-1 and found 50% of ABTS+ scavenging activity at 0.5 mg/mL, a value comparable to that observed in our study with the canthaxanthin from *P. bogoriensis* PH1 (60% at 0.4 mg/mL). A reduction in its antioxidant activity compared to the canthaxanthin standard could be the result of the compound’s instability and susceptibility to degradation during the TLC process.

## 5. Conclusions

This study demonstrated the optimization processes for total carotenoid and canthaxanthin production by *P. bogoriensis* PH1, which was isolated from a hot spring. Flask optimization revealed that carotenoids produced by this strain required a temperature above the optimum for growth, and alkaline pH stress could enhance pigment production. Other factors, such as nitrogen and carbon sources, aeration, and agitation, contributed to pigment production. A two-stage pH control (pH-shift) strategy was designed, in which the culture was grown in pH 7 culture medium until it reached the stationary phase, before shifting the pH to 11 for carotenoid pigment production. Moreover, nutrient optimization resulted in a new culture medium formula, designated Polypeptone Sucrose Yeast Extract (PPSYE) Broth, which contained 2.5% polypeptone and 1% sucrose as the main nitrogen and carbon sources, respectively. Transferring the flask-scale optimized conditions to a bioreactor, an optimized 1 L batch culture process for canthaxanthin production by this strain was developed, which involved cultivation of strain PH1 in PPSYE (pH 7) for 36 h for the growth stage, followed by a pH shift to 11 for 12 h for the canthaxanthin production stage. This process resulted in a 3.12- and 1.61-fold (61%) increase in total carotenoid and canthaxanthin content, respectively. Purified canthaxanthin exhibited strong ABTS^+^ scavenging activity. These findings indicate that *P. bogoriensis* PH1, a thermotolerant hot spring dweller, is a potential candidate for canthaxanthin production. Its alkaline pH-induced pigment production, together with its ability to thrive at elevated temperatures, offers advantages by reducing the risks of microbial contamination and cooling energy demand during cultivation processes. This is appealing for sustainable industrial production and application as a natural pigment source or functional ingredient in food and feed.

## Data Availability

The 16S rRNA sequence of *P. bogoriensis* PH1 is available in the NCBI database (accession no. PX102350).

## Supplementary Materials

The following supporting information can be downloaded at: https://www.mdpi.com/article/10.3390/biology14101334/s1, Figure S1: Growth characteristics of *P. bogoriensis* PH1 determined by measuring OD_600_ every 24 h for 72 h at various pH values (6–12) incubated at 35 °C, and growth characteristics at different incubation temperatures (30–40 °C) in TSYEB medium (pH 11); Figure S2: Effect of various polypeptone concentration to pigment content in *P. bogoriensis* PH1 sources cultured in TSYEB under pH-shift conditions (pH 7»11); Figure S3: Effect of various sucrose concentration to pigment content in *P. bogoriensis* PH1 sources cultured in medium having 2.5% polypeptone as nitrogen source under pH-shift conditions (pH 7»11); Table S1: Antioxidant activities of purified and standard canthaxanthin compared with ascorbic acid and Trolox (DPPH assay).
