# Supplementary material for: Upregulation of Canthaxanthin Biosynthesis by Paracoccus bogoriensis PH1 from Hot-Spring Origin via Sustainable Fermentation Strategy in Laboratory-Scale Bioreactor
Source: Biology (Basel). 2025 Sep 27;14(10):1334. doi: 10.3390/biology14101334 (PMC12561534; doi:10.3390/biology14101334)
Supplement: Supplementary file 1 [file biology-14-01334-s001.zip › Supplementary Figures.pdf]

Supplementary Figures

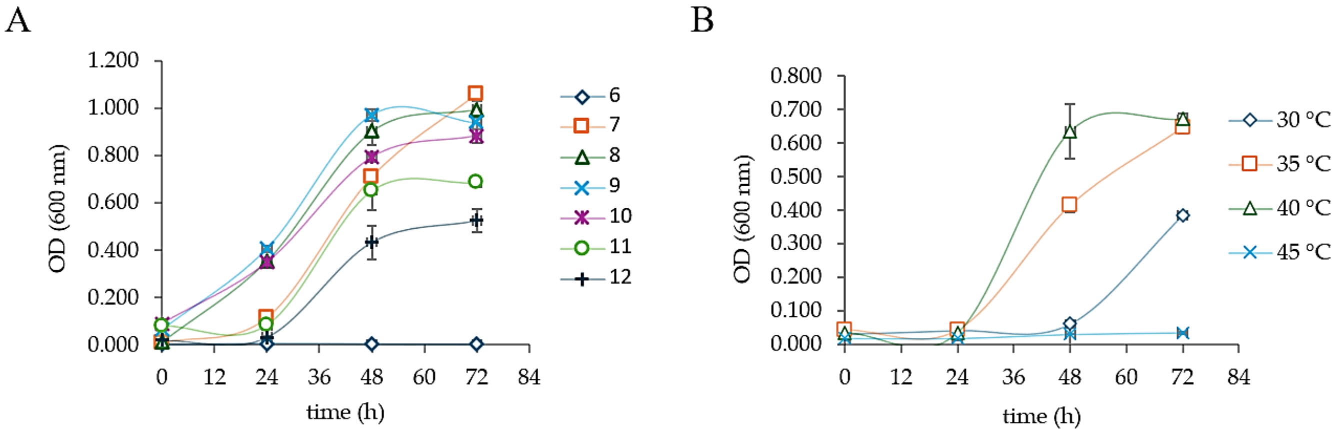

Figure S1. (A) Growth characteristics of *P. bogoriensis* PH1 determined by measuring OD_{600} every 24 h for 72 h at various pH values (6–12) incubated at 35 °C, and **(B)** growth characteristics at different incubation temperatures (30–40 °C) in TSYEB medium (pH 11).

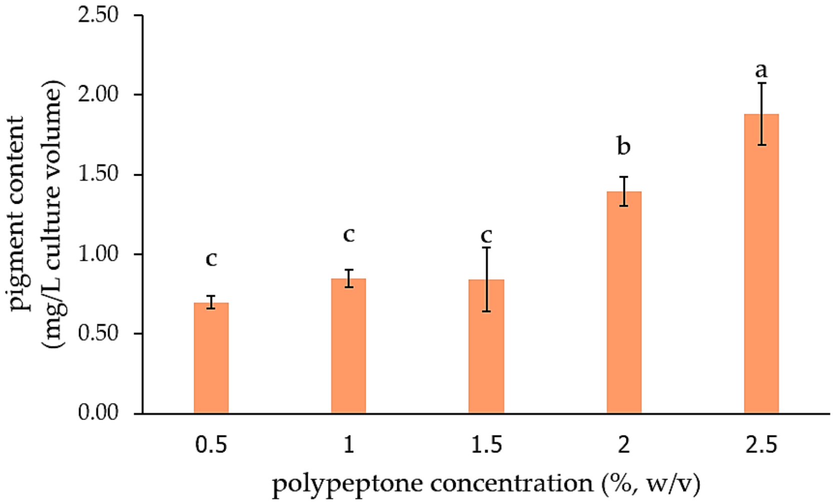

Figure S2. Effect of various polypeptone concentration to pigment content in *P. bogoriensis* PH1 sources cultured in TSYEB under pH-shift conditions (pH 7»11). The pigment content was calculated from a canthaxanthin standard curve. Data are presented as means ± SD. Different letters indicate statistically significant differences (Tukey's HSD test; $p < 0.05$).

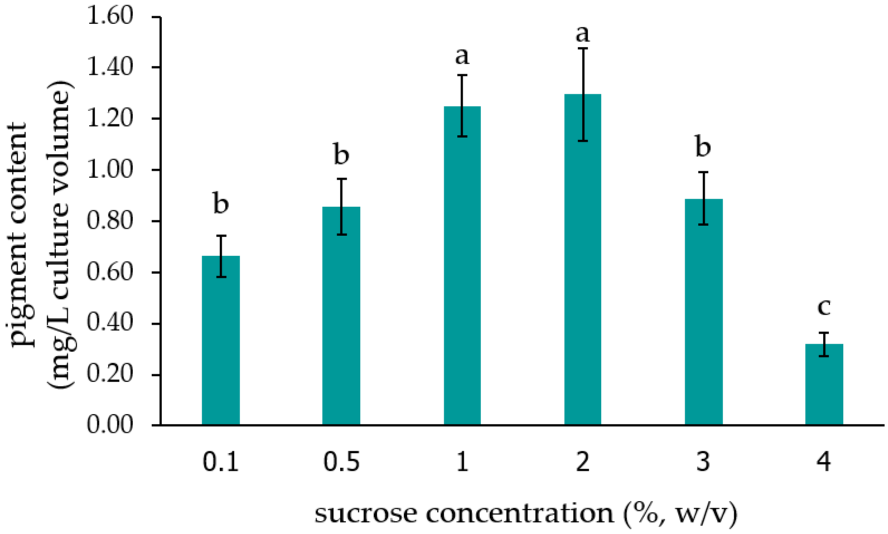

Figure S3. Effect of various sucrose concentration to pigment content in *P. bogoriensis* PH1 sources cultured in medium having 2.5% polypeptone as nitrogen source under pH-shift conditions (pH 7»11). The pigment content was calculated from a canthaxanthin standard curve. Data are presented as means ± SD. Different letters indicate statistically significant differences (Tukey's HSD test; $p < 0.05$).
