# Supplementary material for: Upregulation of Canthaxanthin Biosynthesis by Paracoccus bogoriensis PH1 from Hot-Spring Origin via Sustainable Fermentation Strategy in Laboratory-Scale Bioreactor
Source: Biology (Basel). 2025 Sep 27;14(10):1334. doi: 10.3390/biology14101334 (PMC12561534; doi:10.3390/biology14101334)
Supplement: Supplementary file 1 [file biology-14-01334-s001.zip › Supplementary Table.pdf]

Supplementary Table

Table S1 Antioxidant activities of purified and standard canthaxanthin compared with ascorbic acid and Trolox (DPPH assay)

**Sample**	**DPPH activity at maximum concentration of test (%)**	**IC_{50} value**
Purified canthaxanthin	11.23 ± 0.25	> 0.4 mg/mL
Standard canthaxanthin	11.84 ± 3.89	> 0.4 mg/mL
Ascorbic acid	96.21 ± 1.14	1.97 µg/mL
Trolox	95.48 ± 1.86	2.64 µg/mL

Maximum concentration of test: Purified canthaxanthin (0.4 mg/mL), standard canthaxanthin (0.4 mg/mL), ascorbic acid (4 µg/mL), and Trolox (5 µg/mL).
